# Anti-TNF-α treatment modulates SASP and SASP-related microRNAs in endothelial cells and in circulating angiogenic cells

**DOI:** 10.18632/oncotarget.7858

**Published:** 2016-03-02

**Authors:** Francesco Prattichizzo, Angelica Giuliani, Rina Recchioni, Massimiliano Bonafè, Fiorella Marcheselli, Sabrina De Carolis, Anna Campanati, Katia Giuliodori, Maria Rita Rippo, Francesca Brugè, Luca Tiano, Carla Micucci, Antonio Ceriello, Annamaria Offidani, Antonio Domenico Procopio, Fabiola Olivieri

**Affiliations:** ^1^ Department of Clinical and Molecular Sciences, DISCLIMO, Università Politecnica delle Marche, Ancona, Italy; ^2^ Insititut d'Investigacions Biomèdiques August Pi i Sunyer (IDIBAPS) and Centro de Investigación Biomédica en Red de Diabetes y Enfermedades Metabólicas Asociadas (CIBERDEM), Barcelona, Spain and IRCCS MultiMedica Sesto San Giovanni (MI), Italy; ^3^ Center of Clinical Pathology and Innovative Therapy, National Institute INRCA-IRCCS, Ancona, Italy; ^4^ Department of Experimental, Diagnostic and Specialty Medicine, DIMES, University of Bologna, Bologna, Italy; ^5^ Dermatology Clinic, Department of Clinical and Molecular Medicine, Università Politecnica delle Marche, Ancona, Italy; ^6^ Department of Clinical and Dental Sciences, DISCO, Università Politecnica delle Marche, Ancona, Italy

**Keywords:** miR-146a-5p, miR-126-3p, SASP, HUVEC, replicative senescence, Gerotarget

## Abstract

Endothelial cell senescence is characterized by acquisition of senescence-associated secretory phenotype (SASP), able to promote inflammaging and cancer progression. Emerging evidence suggest that preventing SASP development could help to slow the rate of aging and the progression of age-related diseases, including cancer. Aim of this study was to evaluate whether and how adalimumab, a monoclonal antibody directed against tumor necrosis factor-α (TNF-α), a major SASP component, can prevent the SASP. A three-pronged approach has been adopted to assess the if adalimumab is able to: i) modulate a panel of classic and novel senescence- and SASP-associated markers (interleukin [IL]-6, senescence associated-β-galactosidase, p16/Ink4a, plasminogen activator inhibitor 1, endothelial nitric oxide synthase, miR-146a-5p/Irak1 and miR-126-3p/Spred1) in human umbilical vein endothelial cells (HUVECs); ii) reduce the paracrine effects of senescent HUVECs' secretome on MCF-7 breast cancer cells, through wound healing and mammosphere assay; and iii) exert significant decrease of miR-146a-5p and increase of miR-126-3p in circulating angiogenic cells (CACs) from psoriasis patients receiving adalimumab in monotherapy.

TNF-α blockade associated with adalimumab induced significant reduction in released IL-6 and significant increase in eNOS and miR-126-3p expression levels in long-term HUVEC cultures.

A significant reduction in miR-146a-5p expression levels both in long-term HUVEC cultures and in CACs isolated from psoriasis patients was also evident. Interestingly, conditioned medium from senescent HUVECs treated with adalimumab was less consistent than medium from untreated cells in inducing migration- and mammosphere- promoting effects on MCF-7 cells.

Our findings suggest that adalimumab can induce epigenetic modifications in cells undergoing senescence, thus contributing to the attenuation of SASP tumor-promoting effects.

## INTRODUCTION

The senescence status of stromal cells, including endothelial cells (ECs), plays a major role in inflammaging [1], the low-grade, chronic, and systemic inflammatory condition associated to aging [2]. Cellular senescence is related to the acquisition of a discrete phenotype, the so called senescence-associated secretory phenotype (SASP), characterized by the activation of a pro-inflammatory transcriptional program [3-6]. Accordingly, the pathways involved in SASP acquisition, as the NF-kB and the IL-1/NLRP3 inflammasome pathways are master modulators of the aging rate [7–12]. Notably, removal of senescent cells in animal models, is able to prolong lifespan and healthspan [13]. Evidence that the number of senescent dermal fibroblasts correlates with the presence of some age-related diseases (ARDs) has also been reported in humans [14].

Interventions directed at preventing the adverse effects associated with the SASP are being explored The most promising strategies involve delaying cellular senescence [15, 16]; SASP switch-off [17–20]; and selective removal or killing of existing senescent cells [1, 21]. Even though SASP involves the release of hundreds of molecules [3, 4], like interleukin (IL)-1, IL-6, IL-8, tumor growth factor (TGF)-β, and tumor necrosis factor (TNF)-α, the most common and best characterized [4, 22–26]. Some of these cytokines can induce or reinforce the senescent phenotype by acting in autocrine and paracrine manner, spreading senescence *via* a “bystander effect” [9, 22, 26]. However, TNF-α inhibition in relation to EC senescence and SASP acquisition has not been already extensively explored yet. TNF-α can promote senescence in endothelial progenitor cells [27] and human umbilical vein endothelial cell (HUVEC) cultures [28], and it has well-known adverse effects on endothelial function *in vivo* [29–31]. However the molecular basis for these effects has not been fully elucidated yet.

Here we tested whether TNF-α blockade can reduce the acquisition of the senescent phenotype and/or the SASP by HUVECs, an *in vitro* EC model. TNF-α was inhibited by administration of adalimumab, a monoclonal antibody directed against TNF-α that has been licensed for use in psoriasis [30–34]. To gain insights into the ability of anti-TNF-α treatment to induce epigenetic modifications *in vivo*, microRNAs (miRNAs), key modulators of gene expression, were analyzed in circulating angiogenic cells (CACs) from psoriasis patients treated with adalimumab.

The ability of adalimumab to restrain or delay SASP acquisition was tested by evaluating two senescence/SASP-associated miRNAs (miR-126-3p and miR-146a-5p) and the respective targets (Spred1 and Irak1) [35] as well as a panel of classic senescence- and SASP-associated markers: senescence associated-β-galactosidase (SA-β-Gal) [36], p16/Ink4a [37], plasminogen activator inhibitor 1 (PAI1) [38], endothelial nitric oxide synthase (eNOS) [39] and the prototypical SASP protein IL-6 [3].

Moreover, since the inflammatory tumor microenvironment plays a pivotal role in cancer promotion and progression, and SASP could contribute to tumor growth and tumor cell motility and invasiveness [3, 4, 17], this study also investigates whether anti-TNF-α treatment can modulate the effect of the secretome of senescent ECs on tumor cell motility and on its ability to promote mammosphere formation.

## RESULTS

### Anti-TNF-α treatment of THP-1 cells

**Figure 1:**
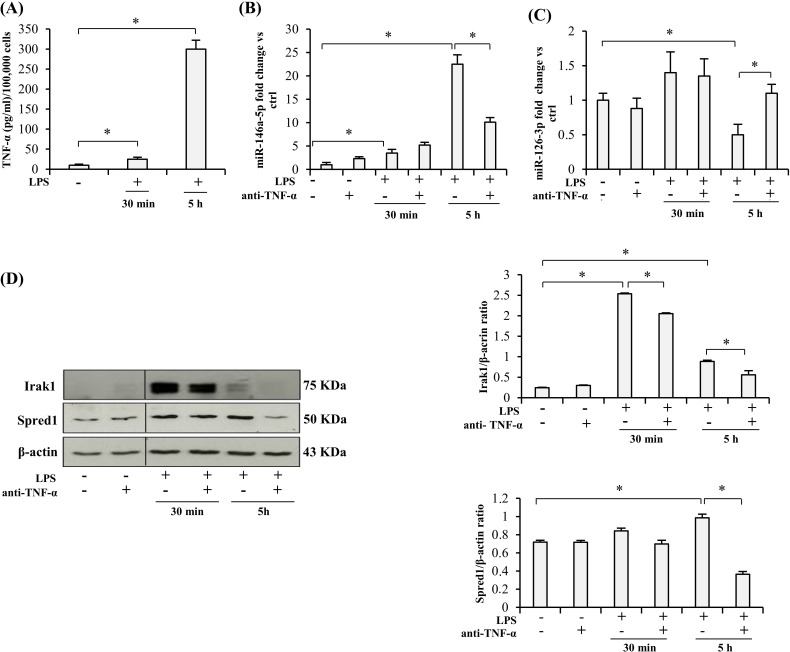
Effect of TNF-α blockade on the expression of miRs and their target proteins in LPS-exposed THP-1 cells **A.** TNF-α release into the culture medium by THP-1 cells after LPS treatment (1 μg/ml), expressed as pg/ml per 100,000 cells. **B.** MiR-146a-5p expression in THP-1 cells after 30 min or 5 h (hours) LPS exposure, with/without 24 h anti-TNF-α pretreatment, measured as fold change *vs* ctrl. C. MiR-126-3p expression in THP-1 cells after 30 min or 5 h LPS exposure, with/without 24 h anti-TNF-α pretreatment, measured as fold change *vs* ctrl. D. Irak1 and Spred1 expression levels and densitometry data in THP-1 cells after 30 min or 5 h LPS stimulation, with/without 24 h anti-TNF-α pretreatment. * Student's *t* test, *p* < 0.05. Data are mean ± S.D. of 3 independent experiments.

Since activated monocytes are major TNF-α producers, and adalimumab is a specific antibody inhibiting its autocrine and paracrine proinflammatory effects, we first tested its ability to modulate miR-146a-5p (an inflamma-miR), miR-126-3p (an endo-miR), and Spred1 and Irak1 (two of their targets) in the human monocytic cell line THP-1 exposed to LPS stimulation for 30 min or 5 h. The levels of the two miRs and the amount of target proteins determined in cells not exposed to LPS were considered as baseline. LPS stimulation induced a significantly increased release of TNF-α that was highest at 5 h, it raised miR-146a-5p expression, and reduced miR-126a-3p levels (Figure 1A, 1B, and 1C). Irak1 protein rose significantly at 30 min whereas Spred1 levels peaked at 5 h, but the latter change was modest (Figure 1D).

Interestingly, pretreatment with anti-TNF-α for 24 h significantly inhibited LPS-induced miR-146a-5p up-regulation and miR-126-3p down-regulation (Figure 1B and 1C), it attenuated the increase in Irak1 levels (Figure 1D), and completely abolished the slight increase in Spred1 protein (Figure 1D).

### Anti-TNF-α treatment of HUVECs

#### 1 Effects of TNF-α inhibition on young and senescent HUVECs

##### Modulation of miR-146a/Irak1 and miR-126-3p/Spred1

**Figure 2:**
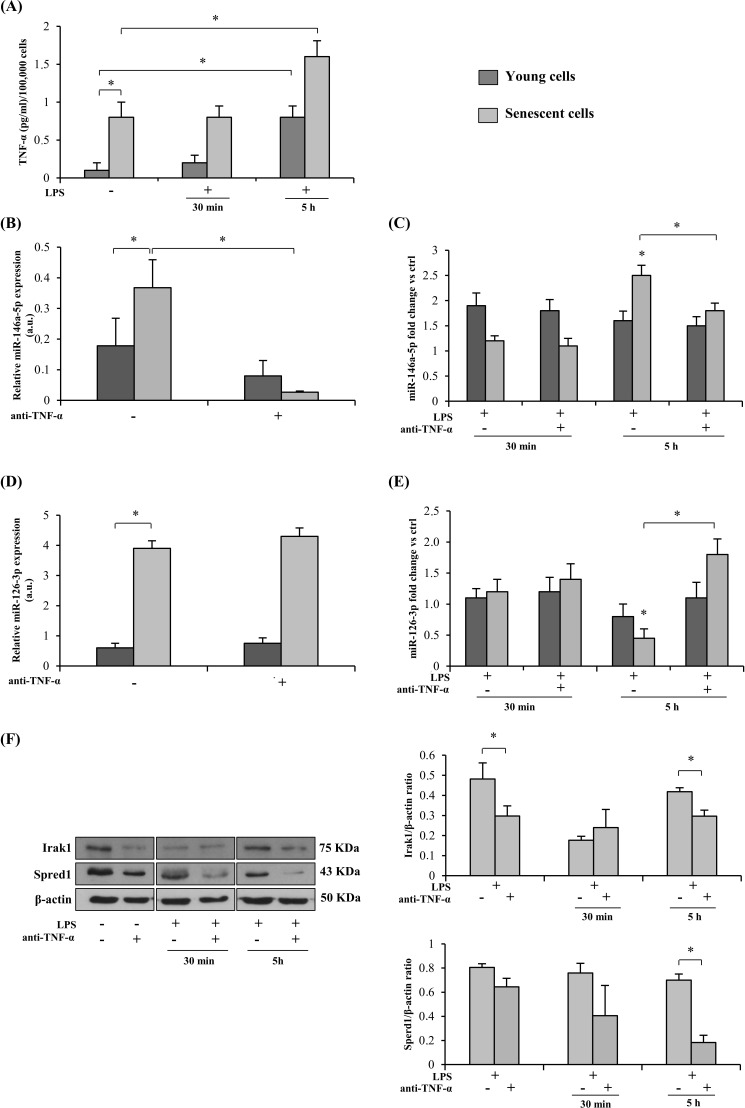
Effect of TNF-α blockade on the expression of miRs and their target proteins in senescent (SA-β-Gal > 50 %) and young (SA-β-Gal < 5 %) HUVECs with and without LPS-stimulation **A.** TNF-α release into the culture medium by LPS-exposed (1 μg/ml) young and senescent HUVECs, expressed as pg/ml per 100,000 cells. **B.** MiR-146a-5p expression in young and senescent HUVECs with/without 24 h anti-TNF-α pretreatment, measured as relative expression (a.u). C. MiR-146a-5p expression in young and senescent HUVECs after 30 min or 5 h LPS stimulation, with/without 24 h anti-TNF-α pretreatment, measured as fold change *vs* ctrl. D. MiR-126-3p expression in young and senescent HUVECs with/without 24 h anti-TNF-α pretreatment, measured as relative expression (a.u). E. MiR-126-3p expression in young and senescent HUVECs after 30 min or 5 h LPS stimulation, with/without 24 h anti-TNF-α pretreatment, measured as fold change *vs* ctrl. F. Irak1 and Spred1 expression and densitometry data in young and senescent HUVECs after 30 min or 5 h LPS stimulation, with/without 24 h anti-TNF-α pretreatment. * Student's *t* test, *p* < 0.05. Data are mean ± S.D. of 3 independent experiments.

Also in the case of HUVECs, LPS stimulation induced release of an increased amount of TNF-α that was highest at 5 h (Figure 2A). Since HUVECs undergoing replicative senescence *in vitro* acquire the SASP, *i.e.* the pro-inflammatory secretory phenotype characterized by increased release of TNF-α and others cytokines (Figure 2A) [15], the inhibitory effect of TNF-α on LPS-treated HUVECs was assayed separately in young and senescent cells. The latter were identified based on the expression of senescence-associated biomarkers, including SASP acquisition (SA-β-Gal > 50 %).

The anti-TNF-α concentration used in our experiments (8 μg/ml), similar to the level measured in the blood of patients treated with adalimumab [40], affected neither the proliferation of young HUVECs (Supplementary Figure 1A) nor the metabolic activity of both young and senescent HUVECs as evaluated by the MTT assay (Supplementary Figure 1B), suggesting that adalimumab does not exert an effective senolytic activity.

MiR-146a-5p and miR-126-3p levels were higher in senescent than in young HUVECs (Figure 2A and 2D). However, while LPS exposure raised miR-146a-5p levels in both sets of cells, miR-126-3p was significantly down-regulated in senescent cells at 5 h, whereas in young cells it was not significantly affected either by LPS or by anti-TNF-α (Figure 2B and 2E), in line with earlier reports [39, 41].

Comparison of miR-146a-5p expression in young and senescent cells after 24 h adalimumab pretreatment highlighted a significant inhibitory effect only in senescent cells, both before and after 5 h LPS exposure (Figure 2B and 2C). Similar results were obtained with different doses of adalimumab (Supplementary Figure 1C).

Irak1 protein levels paralleled the trend of miR-146a-5p expression (Figure 2F).

As regards Spred1, its expression was significantly reduced in senescent cells treated with adalimumab and exposed to LPS (Figure 2F), closely paralleling miR-126-3p expression (Figure 2E).

Neither Irak1 nor Spred1 were significant modulated in young cells (data not shown).

##### Modulation of interleukin IL-6

The effect of adalimumab on IL-6 was investigated because it is the prototypical SASP protein [3, 4]. Adalimumab treatment for 24 h induced a decreased IL-6 release by young HUVECs (SA-β-Gal < 5 %) exposed to LPS stimulation, attenuating the up-regulation due to LPS treatment; in contrast, no significant change was noted in senescent HUVECs (Supplementary Figure 2). Notably, IL-6 release was greater in LPS-untreated senescent HUVECs than in LPS-exposed young cells (Supplementary Figure 2).

#### 2 Effects of TNF-α inhibition on HUVECs undergoing replicative senescence

**Figure 3:**
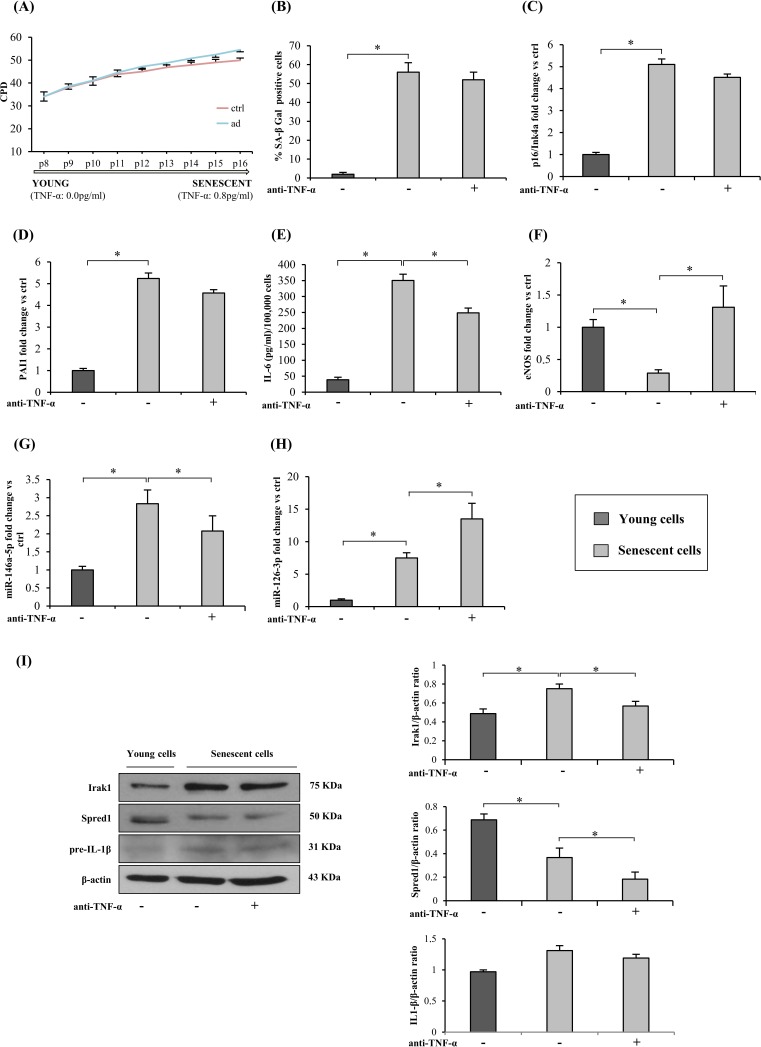
TNF-α blockade and HUVECs replicative senescence and SASP acquisition **A.** Cumulative population doublings (CPDs) of HUVECs exposed to continuous anti-TNF-α treatment and of control cultures from 34 CPDs to complete growth arrest. Y axis, CPD; x axis, number of passages. **B.** Percentage of SA-β-Gal-positive cells at the beginning of the curve (young cells) and at its end (senescent cells), with/without anti-TNF-α treatment. **C.** p16/Ink4a mRNA expression in young and senescent cells with/without anti-TNF-α treatment. Data expressed as fold changes *vs* young cells. **D.** PAI1 mRNA expression in young and senescent cells with/without anti-TNF-α treatment. Data expressed as fold changes *vs* young cells. **E.** IL-6 release (pg/ml per 100,000 cells) into the culture medium by young and senescent cells with/without anti-TNF-α treatment. **F.** eNOS mRNA expression in young and senescent cells with/without anti-TNF-α treatment. Data expressed as fold changes *vs* young cells. **G.** MiR-146a-5p expression in young and senescent cells with/without anti-TNF-α treatment. Data expressed as fold changes *vs* young cells. **H.** MiR-126-3p expression in young and senescent cells with/without anti-TNF-α treatment. Data expressed as fold changes *vs* young cells. **I.** Irak1, Spred1 and IL1β expression and densitometry data (normalized to β-actin) in young and senescent cells with/without anti-TNF-α treatment. * Student's *t* test, *p* < 0.05. Data are mean ± S.D. of 3 independent experiments.

Next, we tested whether continuous TNF-α blockade during replicative senescence reduces senescence/SASP markers in HUVECs. Since TNF-α was not detected in the culture medium of young cells (Figure 2A and Figure 3A), adalimumab was added beginning at 34 CPDs. The treatment failed to reduce the percentage of SA-β-Gal-positive cells (Figure 3A and 3B); in addition, it did not significantly affect the increase of p16/Ink4a and PAI1 expression, two classic markers of EC senescence (Figure 3C and 3D).

Importantly, IL-6 released into the medium was significantly lower in adalimumab-treated cells at the end of the growth curve (Figure 3E), reflecting the reduction of SASP-related cytokines.

As regards eNOS, its down-regulation during replicative senescence was completely abolished by adalimumab (Figure 3F).

Adalimumab induced miR-146a-5p and Irak1 down-regulation (Figure 3G and 3I) as well as increased miR-126-3p and decreased Spred1 expression (Figure 3H and 3I). Western blot analysis showed unchanged cellular IL-1β protein levels in both young and senescent HUVECs (Figure 3I) and undetectable IL-1β levels in the culture medium (data not shown).

### Effects of TNF-α inhibition on the SASP bystander effect

**Figure 4:**
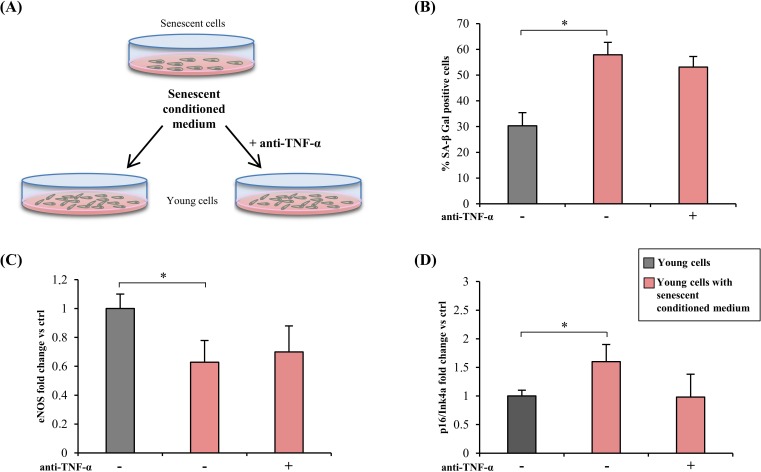
TNF-α blockade and bystander effect of HUVECs SASP **A.** Drawing showing the experimental design. Conditioned medium from senescent cells (SA-β-Gal > 50 %) was mixed with 1/3 of fresh medium (with 30 % serum) and used to treat young cells (SA-β-Gal < 5 %) for 2 weeks, with/without anti-TNF-α treatment. Conditioned medium from young cells was used as control. **B.** Percentage of SA-β-Gal positive cells after 2 week exposure to conditioned media, with/without anti-TNF-α treatment. **C.** and **D.** eNOS and p16/Ink4a expression in cells treated with conditioned medium, with/without anti-TNF-α treatment. Data expressed as fold change *vs* control cells. * Student's *t* test, *p* < 0.05. Data are mean ± S.D. of 3 independent experiments.

To test the ability of TNF-α inhibition to induce a bystander effect, young HUVECs were treated for 2 weeks with conditioned medium from senescent HUVECs (SA-β-Gal > 50 %) mixed with 1/3 fresh medium (with 30 % serum), with or without adalimumab (Figure 4A). Conditioned medium from young cells was used as control.

Exposure of young HUVECs to conditioned medium from senescent cells induced a doubling of SA-β-Gal-positive cells, including adalimumab-treated ones (Figure 4B). The medium also induced eNOS down-regulation and p16/Ink4a up-regulation; neither effect was significantly reversed by anti-TNF-α (Figure 4C and 4D).

### Effects of TNF-α inhibition in the secretome of senescent HUVECs on tumor cell migration

**Figure 5:**
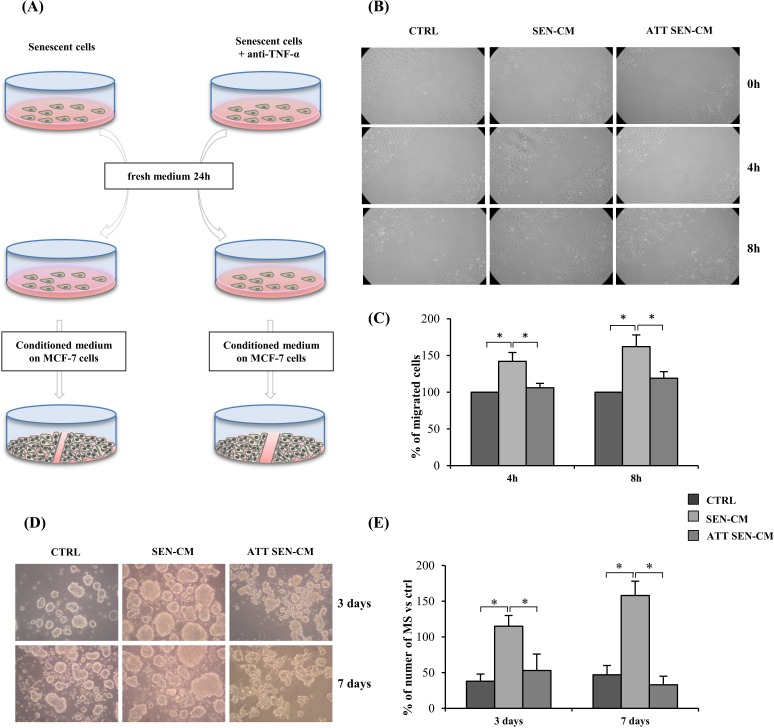
Anti-TNF-α treatment effect on pro-motility activity and mammosphers (MS) promotion of HUVECs secretome on MCF-7 tumor cell **A.** Drawing showing the experimental design. Senescent cells exposed to anti-TNF-α long-term treatment and senescent control cells were switched to fresh medium for 24 h and then their conditioned medium was mixed with 1/3 fresh DMEM. These mixtures, senescence conditioned medium (SEN-CM), and anti-TNF-α treated senescence conditioned medium (ATT SEN-CM) were used to treat MCF-7 in a wound healing assay. Conditioned medium from young cells was mixed with DMEM and used as control. **B.** Light microscopic photographs showing MCF-7 migration in the wound healing assay 0, 4 and 8 h after treatment with SEN-CM or ATT SEN-CM. **C.** Percentage of migrating cells after 4 and 8 h exposure to SEN-CM and ATT SEN-CM. Data expressed as percent of control. **D.** Representative pictures of MCF7-derived mammosphers (MS) promotion induced by conditioned media obtained from young cells (CTRL-CM), senescent cells (SEN-CM), and anti-TNF-α treated senescent cells (ATT SEN-CM). **E.** Quantification of MCF7-derived mammospheres in presence of different conditioned media. Data are presented as number of MS per well, at 3 and 7 days. Data are mean ± S.D. of 3 independent experiments. Scale bar 50 μm. * *p* < 0.05. * Student's *t* test, *p* < 0.05. Data are mean ± S.D. of 3 independent experiments.

Senescent cells exert a tumorigenic effect [3]. To test whether long-term anti-TNF-α treatment reduces the cell non-autonomous functions of the endothelial SASP, a wound healing assay was conducted using MCF-7 breast cancer cells and A549 lung cancer cells cultured in presence of conditioned medium from senescent HUVECs. Notably, the MCF-7 cell line is responsive to IL-6 [41, 42]. Senescent cells subjected to long-term adalimumab treatment and untreated were placed in fresh medium for 24 h and their conditioned medium was mixed with 1/3 fresh DMEM. These mixtures, senescence conditioned medium (SEN-CM), and anti-TNF-α treated senescence conditioned medium (ATT SEN-CM) were used to treat MCF-7 cells in the wound healing assay (Figure 5A). Conditioned medium from young cells mixed with DMEM was used as control.

SEN-CM strongly promoted MCF-7 migration whereas ATT SEN-CM did not (Figure 5B and 5C), clearly indicating that long-term anti-TNF-α treatment reduces the HUVEC SASP. In contrast, migration of A549 cells was not enhanced by SEN-CM (data not shown), suggesting that different tumors respond differently to the SASP.

### Effects of TNF-α inhibition in the secretome of senescent HUVECs on mammospheres assay

The mammosphere assay allows to propagate a population of putative cancer-stem-cells as floating spheroids [43–45]. This assays represents a useful surrogate test to screen the tumorigenic and metastatic potential of breast cancer cells [43].

The same conditioned media obtained from young cells (CTRL-CM), senescent cells (SEN-CM), and anti-TNF-α treated senescent cells (ATT SEN-CM) used to treat MCF-7 cells in the wound healing assay were used for MCF-7 mammospheres (MS) assay (Figure 5D and 5E). Primary MS formation was assessed after 3 and 7 days.

SEN-CM strongly promoted the formation of MCF-7-derived mammospheres, whereas in ATT SEN-CM this promotion is significantly reduced both at 3 and at 7 days (*p* > 0.05) (Figure 5D and 5E).

### Effects of TNF-α inhibition on CACs from psoriatic patients

**Figure 6:**
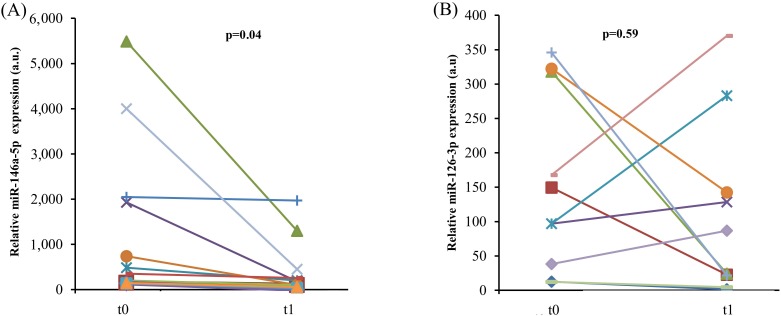
*In vivo* anti-TNF-α treatment and endothelial senescence-associated miR expression levels in psoriatic patients MiR-146a-5p **A.** and miR-126-3p **B.** expression in CACs from 13 psoriasis patients before and after 3 month anti-TNF-α monotherapy with adalimumab. P from Student's t test for paired samples.

Psoriasis patients show elevated circulating levels of TNF-α compared with healthy subjects, and psoriasis has been associated with an increased incidence of ARDs [46, 47]. Given that CACs are monocytes capable of releasing pro-angiogenic cytokines, CACs from 10 psoriatic patients, receiving adalimumab after a wash-out period, were analyzed to establish whether anti-TNF-α monotherapy for 3 months is able to reduce miR-126-3p and miR-146a-5p expression. TNF-α inhibition affected miR-146a-5p levels (Figure 6A), as also noted in the experiments with THP-1 cells and HUVECs, suggesting that the epigenetic modulation of inflammatory pathways induced by adalimumab *in vitro* resembles the modulation induced *in vivo*. Notably, miR-126-5p expression was not significantly affected in psoriasis patients, suggesting that the anti-inflammatory effects of adalimumab prevail on its proangiogenic effects (Figure 6B).

## DISCUSSION

Selective removal of senescent cells and SASP attenuation are emerging as major goals of aging research. Avoiding the adverse effect associated with the SASP has a great potential to enhance successful aging [1]. Here we document that inhibition of TNF-α activity in ECs undergoing replicative senescence attenuated the SASP, as demonstrated by the down-regulation of IL-6, the suppression of miR-146a-5p and the increase of miR-126-3p, respectively an inflamma-miR and an endo-miR, and their targets. Importantly, anti-TNF-α treatment also induced eNOS up-regulation, suggesting an enhanced endothelial function.

Interestingly, these significant effects induced in HUVECs undergoing replicative senescence were not associated with significant decrease of classic senescence biomarkers, such as SA-β-Gal, p16/Ink4a, and PAI1.

Some studies have described the possibility of dissociating experimentally the SASP from senescence [9, 17]. Although a number of reports have shown that SASP modulation influences the rate of senescence [9, 22, 48], differences have been observed depending on the cytokines involved [17]. In our experimental model, *i.e.* HUVECs undergoing replicative senescence, the number of senescent cells was not significantly affected by continuous anti-TNF-α treatment, suggesting that TNF-α is not closely associated with the arrest of replicative growth. Although it has been demonstrated that IL-1 or TGF-β blockade can attenuate SASP spread in different senescence models [9, 22], data on anti-TNF-a treatment were scarce and inconclusive [27, 28]. The present findings now indicate that anti-TNF-α treatment can restrain the SASP without significantly affecting senescence signal transmission, either autocrine or paracrine. Notably, our experimental model involved blocking the TNF-α spontaneously produced by ECs undergoing senescence, which never exceeds 1 pg/ml per 100,000 cells, whereas in other experimental models cells have been treated with greater (up to 10 fold) amounts of TNF-α [27]. Our data demonstrate that anti-TNF-α treatment in the physiological range can reduce some of the cell-non-autonomous effects of the SASP; in particular, conditioned medium from senescent cells treated with adalimumab reduced the migration rate of MCF-7 breast cancer cells and strongly attenuated the mammospheres promoting effect of the senescent HUVECs secretome, suggesting a decreased pro-tumorigenic and pro-metastatic behavior of the SASP under TNF-α blockade condition.

These findings lend support to the hypothesis that the SASP does not need to be completely suppressed to obtain significant effects, or to attenuate the onset/progression of ARDs, including cancer [17].

TNF-α plays a large role in EC inflammation during aging [29]. Treatment with adalimumab reduces the levels of pro-inflammatory circulating cytokines and improves flow-mediated dilation [30, 31, 47], two well-established markers of endothelial aging [49]. Therefore, TNF-α modulation could have a key role both in aging rate and in ARD development and progression [47, 50].

Main limit of our study is that it refers only to the effect of TNF-α on SASP. A number of evidence suggest that several drugs, nutrition interventions and life-style modifications are able to modulate SASP, mainly through the activation of Toll-like receptors (TLRs) and NF-κB pathway activation [51]. SASP modulation should be more extensively investigated through other therapeutic strategies, such as agonist/antagonist of TLRs, focusing on the related decoy receptors and their miRNAs targets

In conclusion, anti-inflammatory treatments capable of restraining the SASP could contribute to delay ARD onset and progression, especially in patients with an established chronic inflammatory background [1, 8, 52–54]. Clearly, TNF-α inhibition has too many side effects to be administered as a clinical anti-aging treatment in old patients [27, 40, 47]. However, the present findings are in line with earlier reports [9, 17] that it is possible to dissociate the SASP from senescence, and encourage the search for substances, synthetic or natural, that not only suppress but also restrain the SASP.

Our data add a piece to the complex puzzle of inflammaging, furthering our knowledge of the mechanisms controlling the SASP in ECs [52] and the associated chronic inflammation that can promote the development and progression of the major ARDs [8, 54].

## EXPERIMENTAL PROCEDURES

### Anti-TNF-α and LPS treatment

After testing different pharmacologically appropriate adalimumab doses in BrDU and MTT assays (Supplementary Figure 1), 8 μg/ml of the human TNF-α inhibitor (Humira, Abbott, Lake Forest, IL, USA) was used in both short- and long-term experiments. This is the concentration commonly found in patient sera following injection of 0.8 ml adalimumab at a concentration of 50 mg/ml [40]. A random IgG at the same dose was always added to control cultures to avoid phenomena involving Fc receptor.

Lipopolysaccharide (LPS; Sigma-Aldrich, Taufkirchen, Germany) was added (1 μg/ml) to the medium as appropriate for the short-term experiments, *i.e.* 30 min and 5 h.

### HUVEC and THP-1 culture

HUVECs derived from 3 donor pools were purchased from Clonetics (Lonza, Basel, Switzerland) and cultured in EGM-2 endothelial growth medium (Lonza). Briefly, fresh cells were seeded at a density of 5000/cm^2^ in T 75 flasks (Corning Costar, Sigma Aldrich, St. Louis MO, USA); the medium was changed at 48 h intervals. Cultures reached confluence after 6-7 days, as assessed by light microscopic examination, and were passaged weekly. After trypsinization and before replating, harvested cells were counted using a hemocytometer. Replicative senescence was studied by culturing cells up to the 15/16^th^ passage. Cumulative population doubling (CPD) was calculated as the sum of all PD changes. Cells were divided into young (SA-β-Gal < 5 %) and senescent (SA-β-Gal > 50 %).

SA-β-Gal activity was assessed as described previously [13].

Human monocytic THP-1 cells were purchased from ATCC (Rockville, MD, USA) and maintained in RPMI-1640 medium supplemented with 10 % heat-inactivated fetal bovine serum, 1 % penicillin/streptomycin, and 1 % L-glutamine (all from Euroclone, Milano, Italy).

### Protein extraction and immunoblotting

Cells were washed twice in cold phosphate buffered saline (PBS). Total protein was extracted using RIPA buffer (150 mMNaCl, 10 mM Tris, pH 7.2, 0.1 % SDS, 1.0 % Triton X-100, 5 mM EDTA, pH 8.0) containing a protease inhibitor cocktail (Roche Applied Science, Indianapolis, IN, USA). Protein concentration was determined using Bradford Reagent (Sigma-Aldrich, Milano, Italy). Total protein extracts (40 μg) were separated by 10 % SDS-PAGE and transferred to PVDF membranes (Bio-Rad, Hercules, CA, USA). Membranes were incubated overnight with primary anti-Spred1 antibody diluted 1:1000 (Thermo Scientific, Pierce Biotechnology, Rockford, IL, USA), anti-Irak1 antibody diluted 1:250 (MBL International Corporation, Nagoya, Japan), and anti-IL-1β antibody diluted 1:1000 (Cell Signaling Technology, Beverly, MA, USA); subsequently they were incubated with a secondary antibody conjugated to horseradish peroxidase for 1 h at room temperature. Immunoreactive proteins were visualized using ECL Plus chemiluminescence substrate (GE Healthcare, Pittsburgh, PA, USA). Membranes were incubated with anti β-actin diluted 1:10,000 (Santa Cruz Biotechnology, Santa Cruz, CA, USA) as an endogenous control.

### Total RNA extraction from HUVECs

Total RNA enriched in miRNA species was purified from HUVECs using RNA purification kit according to the manufacturer's instructions (Norgen Biotek, Thorold, Canada).

### MiRNA quantification by RT q-PCR

MiRNA expression was quantified using a modified real-time approach using the TaqMan miRNA RT kit and a miRNA assay (Applied Biosystems, Foster City, CA, USA). Briefly, total RNA was reverse-transcribed with a TaqMan MicroRNA RT kit. The 5 μl RT reaction volume contained 1 μl of each miR-specific stem-loop primer, 1.7 μl of input RNA, 0.4 μl of 10 mM dNTPs, 0.3 μl reverse transcriptase, 0.5 μl 10x buffer, 0.6 μl RNAse inhibitor diluted 1:10, and 0.5 μl H_2_O. The mixture was incubated at 16°C for 30 min, at 42°C for 30 min, and at 85°C for 5 min. Quantitative real-time PCR was subsequently performed. The 5 μl PCR reaction volume included 0.25 μl 20x TaqMan MicroRNA Assay, which contained the PCR primers and probes (5′-FAM), 2.75 μl 2x TaqMan Universal Master mix no UNG (Applied Biosystems), and 2.00 μl RT product. The reaction was first incubated at 95°C for 2 min, followed by 40 cycles of 95°C for 15 sec and 60°C for 1 min. Data were analyzed with Real Time PCR Opticon Monitor version 2 (MJ Research, Bio-Rad) with automatic Ct setting for adjusting the baseline and threshold for Ct determination.

MiR expression in HUVECs was evaluated using RNU44 as the reference; for miR expression in CACs, RNU 6b was used as the reference. Each reaction was performed in duplicate.

### mRNA expression

For mRNA gene expression, total RNA was reverse-transcribed using RT^2^ First Strand Kit (Norgen Biotek) according to the manufacturer's instructions. The cDNA thus obtained was used for subsequent quantitative real-time PCR (qPCR). qPCR reactions were conducted on a MyiQ Single Color Real-Time PCR Detection System (Bio-Rad) in a 15 μl total reaction volume using iQ^TM^ SYBR Green Supermix (Bio-Rad). The mRNA expression of the genes of interest was calculated with reference to three reference genes, β-actin, β2M, and HPRT1. Primer concentration was 300 nM for β-actin and HPRT1 and 400 nM for the other primers. Each reaction was run in duplicate and always included a no-template control. All primers have been described previously [55].

The qPCR reaction was programmed to start with a 3 min denaturation step at 95°C for polymerase activation followed by 40 cycles of 15 sec denaturation at 95°C and 30 sec of annealing/extension at 60°C, during which fluorescence was measured. Next, a melting curve was constructed by raising the temperature from 55°C to 95°C in sequential 0.5°C steps for 6 sec. PCR efficiencies were all between 90 % and 110 % and were taken into account when calculating Ct values. mRNA quantification was assessed using the 2^−DDCt^ method.

### Cell viability assay

The (3-(4,5-dimethylthiazol-2-yl)-2,5-diphenyltetrazolium bromide (MTT) assay was used to test cell viability. Cells were grown in 96-well plates at a density of 2×103 cells/well. After 18 h they were washed with fresh medium then treated with differential doses of adalimumab. After 24 h pretreatment, 100 μl MTT (1 mg/ml) was added and incubated for 4 h; the formazan salt that formed was solubilized by adding 200 μl dimethyl sulfoxide and its amount was determined by measuring optical density at 540 nm using a microplate reader (MPT Reader, Invitrogen, Milano, Italy).

### Cytokine production

Culture supernatants were collected at the end of each incubation, centrifuged, and stored at −20°C until use in the assays. IL-6 and TNF-α concentrations were measured using a commercially available, high-sensitivity ELISA kit (Invitrogen) or a 4 custom cytokine multiplex (Tema Ricerca, Castenaso, Bologna).

### BrDU assay

DNA Cell proliferation ELISA, BrdU (colorimetric) was purchased from Roche Diagnostics (Mannheim, Germany). Cells were plated in triplicate at a density of 5×103/cm^2^ in 96-well plates and treated with various doses of adalimumab; BrdU labeling solution was then added and plates were read after 24 h incubation according to the manufacturer's instructions.

### Psoriasis patients

A total of 10 psoriatic patients were enrolled in the study. The mean age was 53 ±14 years, 8 males and 2 females. The study protocol was approved by the Ethics Committee of UNIVPM (Ancona, Italy) and all enrolled patients provided a written informed consent. All patients received 3 months of adalimumab monotherapy after a 12 weeks-wash-out period from previous therapy.

### CAC isolation and RNA extraction

CACs were isolated from approximately 14 ml heparinized peripheral blood. Peripheral blood mononuclear cells (PBMCs) were isolated by density-gradient centrifugation with Ficoll (Ficoll-Paque™ PLUS, GE Healthcare Bio-Sciences Uppsala, Sweden) within 2 h of collection. Then 5×106 PBMCs were plated on 24-well fibronectin-coated plates (BD Biosciences, Mountain View, CA, USA) and maintained in endothelial basal medium (EBM; Clonetics-Lonza, Walkersville, MD USA) supplemented with EGM SingleQuots and 20 % fetal calf serum for 4 days. After 4 days in culture, non-adherent cells were removed by washing in PBS, whereas adherent cells were lysed directly in the culture wells.

The CAC phenotype was confirmed by cellular uptake of acetylated LDL (DiI-acLDL) and binding of FITC-conjugated lectin from *Ulex europaeus* (UEA-1) by fluorescence microscopy. Briefly, to detect DiLDL uptake, cells were incubated with DiLDL (2.4 μg/ml) (Molecular Probes, Eugene, OR, USA) at 37°C for 2 h. To detect UEA-1 binding, cells were fixed with 2 % paraformaldehyde for 15 min and incubated with FITC-labeled UEA-1 (10 μg/ml) (UEA-1, Sigma, St. Louis, MO, USA) for 1 h. Double-stained cells positive for both UEA-1 and DiLDL were regarded as CACs. RNA was purified according to the instruction manual of the Total RNA Extraction kit (Norgen Biotek).

### Cultures and generation of MCF7 mammospheres (MS)

MCF7 were grown in RPMI 1640 +10% FBS medium. MCF7-derived mammospheres (MS) were obtained by plating 2500 cells into 3-cm^2^ low-attachment wells (Corning, NY, USA) filled with mammary epithelial growth medium (MEGM), supplemented with MEGM bullet kit (Lonza Ltd, Basel, Switzerland). Primary MS formation was assessed after 3 and 7 days and photographed using a inverted microscope (Olympus CKX41, digital cameras Olympus C-5060, Japan). Only MS with an apparent diameter of ≥50 μm were scored for statistical analysis, as previously described [43, 44].

### Statistical analysis

Data are mean ± standard deviation (S.D.) of at least three independent experiments. Student's *t* test was applied to determine differences between samples. The *t* test for paired samples was used to determine differences in miRNA expression in CACs before and after adalimumab treatment. *P* values < 0.05 were considered significant.

## SUPPLEMENTARY MATERIAL FIGURES


