# Supplementary material for: Anti-TNF-α treatment modulates SASP and SASP-related microRNAs in endothelial cells and in circulating angiogenic cells
Source: Oncotarget. 2016 Mar 2;7(11):11945–58. doi: 10.18632/oncotarget.7858 (PMC4914260; doi:10.18632/oncotarget.7858)
Supplement: Supplementary file 1 [file oncotarget-07-11945-s001.pdf]

Anti-TNF-α treatment modulates SASP and SASP-related microRNAs in endothelial cells and in circulating angiogenic cells

Supplementary Material

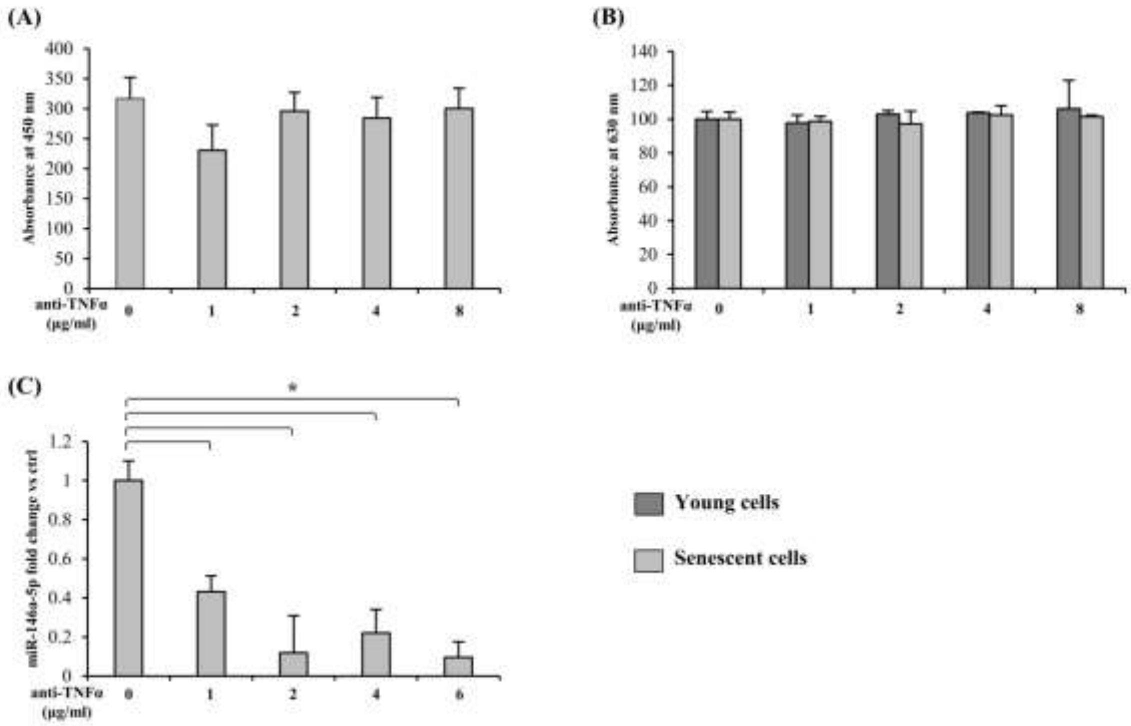

Supplementary Figure 1: Dose-dependent Adalimumab effects on young and senescent ECs

A. A range of adalimumab doses, that are found in patients treated with the drug, failed to affect young EC proliferation.

B. A range of adalimumab doses, that are found in patients treated with the drug, failed to exert cytotoxic effects on young and senescent ECs, as measured with the MTT assay.

C. A range of adalimumab doses, that are found in patients treated with the drug, gave comparable results in terms of miR-146a expression in senescent ECs after 24 h treatment. Data expressed as fold changes *vs* control. Data are mean ± S.D. of 3 independent experiments.

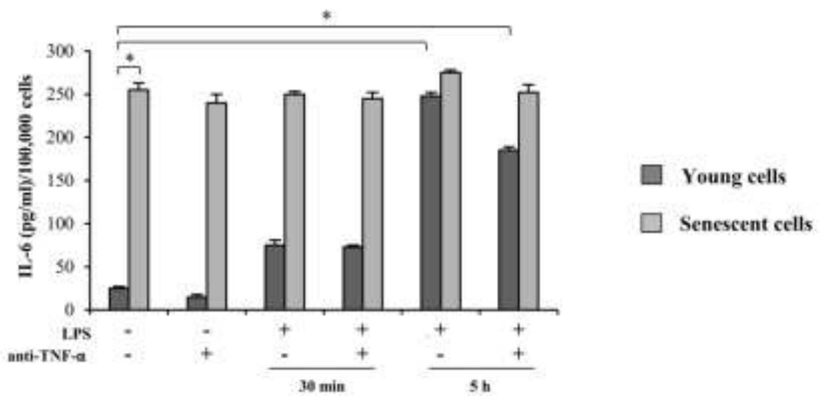

Supplementary Figure 2: Anti-TNF-α treatment and IL-6 release in young and senescent ECs exposed to LPS. IL-6 release into the culture medium by young and senescent ECs exposed to LPS (1 μg/ml), with/without 24 h anti-TNF-α treatment. Data are expressed as μg/ml per 100,000 cells. Data are mean ± S.D. of 3 independent experiments.
